# Difference of efficacy between Laparoscopic Modified Soave operation and Open Radical Resection in the treatment of Hirschsprung’s disease

**DOI:** 10.12669/pjms.336.13220

**Published:** 2017

**Authors:** Yali Tian, Tianting Shi, Fang Wang, Yurui Wu

**Affiliations:** 1 Yali Tian, Department of Pediatric Surgery, Binzhou People’s Hospital, Shandong 256610, China; 2 Tianting Shi, Department of General Surgery, Binzhou People’s Hospital, Shandong 256610, China; 3 Fang Wang, Department of General Surgery, Binzhou People’s Hospital, Shandong 256610, China; 4 Yurui Wu, Department of Minimally Invasive Surgery, Qilu Children’s Hospital of Shandong University, Shandong, 250022, China

**Keywords:** Hirschsprung’s disease, Laparoscope, Modified Soave operation, Open radical resection

## Abstract

**Objective:**

To analyze and compare the efficacy of laparoscopic modified Soave operation and open radical resection in the treatment of Hirschsprung’s disease.

**Methods:**

Two hundred and sixteen children who suffered from Hirschsprung’s disease and were admitted into the hospital from June 2015 to December 2016 were selected as research subjects. They were grouped into an observation group in which patients were treated by laparoscopic modified Soave operation and open radical resection and a control group in which patients were treated by open radical resection. The clinical efficacy and complications of the two groups were observed, and the defecation function was also evaluated.

**Results:**

Operation indicators such as the operation time, time to recovery of intestine peristalsis, intraoperative blood loss and pain score of the observation group were superior to those of the control group, and the difference had statistical significance (P<0.05). The mean arterial pressure (MAP) and heart rate (HR) of the observation group were lower than those of the control group at all time points after operation, and the difference suggested statistical significance (P<0.05). The postoperative complications of the observation group were less than those of the control group. The follow-up results demonstrated that the excellent and good rate of Kelly score of the observation group was 81.5%, higher than 61.1% in the control group.

**Conclusion:**

Laparoscopic modified Soave operation has definite efficacy in the treatment of Hirschsprung’s disease, and the treatment is featured by high safety and few complications, which is beneficial to the recovery of defecation function; hence laparoscopic modified Soave operation is worth clinical promotion.

## INTRODUCTION

Pediatric Hirschsprung’s disease which manifests as repetitive abdominal distention is commonly seen in clinics, and its cause is the lack of ganglion cells in intestinal canal. Investigations have suggested that^1^,^2^, the number of Hirschsprung’s disease children who received treatment showed a sustainable growth in recent years, and its morbidity has ranked the second place among various digestive tract abnormality. Open surgery is a common therapy for Hirschsprung’s disease; however, it has defects such as serious trauma, long postoperative recovery time and high incidence of complications.^3^

Soave operation has gained an extensive application in clinics since transanal Soave operation was reported being used for treating Hirschsprung’s disease abroad in 1988. Soave operation which was modified based on Swenon operation can complete submucosal separation in the rectum, i.e., pulling the aganglionic colon out, avoiding the injury on the deep tissue structure of the pelvic cavity. Smith et al. initially reported the application of laparoscope in Duhamel megacolon radical operation in 1994.^4^ Georgenson et al. reported the clinical application of laparoscopic Soave operation in the treatment of megacolon.^5^

Since then, the application of laparoscopic radical operation for treating pediatric Hirschsprung’s disease became more and more frequent, and laparoscopic operation is gradually improved. Currently, laparoscopic Soave operation is frequently used, which changes the routine of the traditional operation, effectively reduces injury on patients and improves surgical tolerance.^6^ This study investigated the clinical efficacy of modified Soave operation under the assistance of laparoscope by analyzing the Hirschsprung’s disease patients who were admitted into Binzhou People’s Hospital, Shandong, China, aiming to provide a reference for clinical treatment.

## METHODS

Two hundred and sixteen children who were confirmed as Hirschsprung’s disease by clinical examination, rectum mucosa biopsy and X-ray barium enema examination according to Practical Paidonosology^7^ and were admitted to Binzhou People’s Hospital, Shandong, China between June 2015 and December 2016 were selected. It included 187 cases of common type and 29 cases of long-segment type. They were divided into two groups randomly, i.e., an observation group in which patients were treated by laparoscopic modified Soave operation and a control group in which patients were treated by open radical resection. All the children had first time for irschsprung’s disease surgery. In the observation group (n=108), there were 78 males and 30 females; they aged from two months to two years (average (9.3±1.5) months) and had an average weight of (8.2±1.9) kg. In the control group (n=108), there were 86 males and 22 females; they aged from 2.2 months to two years (average (9.7±1.4) months) and had an average weight of (8.3±2.1) kg. The differences of gender constituent ratio, age and weight between the two groups had no statistical significance (P>0.05). This study has been reviewed and approved by the ethics committee of our hospital. The parents of the children were informed with the study content and were willing to coordinate with doctors.

All the children were given reflux enema for 10 to 14 days before surgery. The nutrition condition was improved. Relevant complications were treated. Moreover they were given low residue diet for three days and orally administrated intestinal antibiotics. Patients in the observation group were treated by laparoscopic modified Soave operation according to the following procedures. The patient took a supine position, with feet lifted, and then underwent general anesthesia by means of tracheal intubation. The abdomen, private parts and lower limbs were disinfected and then covered by sterile sheets. The position of the children was adjusted during surgery. The monitor was placed on the left side of the operating table. A veress needle was inserted along the lower edge of navel to establish CO_2_ pneumoperitoneum; pressure was kept at 8~10 mmHg. Then a 5-mm Tocar needle was punctured; after a 30° laparoscope was inserted, the Trocar needle was punctured at the left and right middle and lower abdomen; a handling tongs was inserted to probe the abdominal cavity and confirm the area of lesions. An ultrasound knife was used to remove intestinal canal, dissociate mesocolon, and interrupt mesenterium at grade II vascular arch to ensure the dissociative colon had no tension when being pulled through. Till inlet of the pelvic cavity, the rectum was dissociated till peritoneal reflection; the upper part of lateral rectal ligments must be dissociated. If bleeding was not observed, the pneumoperitoneum was relieved. Next was operation on the perineum. The modified Soave operation was performed on the anus. Eight stitches were sutured around the anus to fully expose anal tube. At the sites where were one cm above the dentate line of rectal posterior wall and 2~3 cm above the dentate line of anterior rectal wall respectively, rectum mucosa and musculi sphincter ani internus were cut apart obliquely. Antetheca was separated along internal and external sphincter muscle clearance till muscular sheath was cut apart at the level of peritoneal reflection; then muscular sheath was removed. Pneumoperitoneum was re-established. The dissociated colon and rectum were pulled out of the body via anorectal muscular sheath under the monitoring of the laparoscope. The diseased intestinal canal was removed. Then fast frozen pathological examination was performed to confirm the presence of nerve cells. Proximal colon was sutured with the cut edge of rectum mucosa. The anal tube was indwelled in the anus to observe CO_2_ in the abdominal cavity and exhaust.

Whether the pulled colon had distortion, bleeding or internal hernia was confirmed before the end of surgery. The pneumoperitoneum was relieved; CO_2_ in the abdominal cavity was discharged; then the cannula was removed, and the small incision was sutured.

### Observation indicators

The surgical indicators such as duration of operation, intraoperative blood loss, time to recovery of postoperative peristalsis and pain score and postoperative complications were observed and recorded accurately. Postoperative pain was scored using 10-point objective pain grading scale. Mean arterial pressure (MAP), heart rate (HR) and blood oxygen saturation (SpO_2_) were monitored before operation (T0) and 8 hour, 16 hour and 24 hour after operation (T1, T2 and T3). One-year follow up was carried out. The recovery of defecation was evaluated in the aspects of frequency, fecal character, defile feces, rectal sensation and bowel control capacity using Kelly score scale. A score of 14 points was determined as excellent, a score of 10~13 points was determined as good, a score of 5~9 points was determined as general, and a score of 5 point was determined as poor.

### Statistical analysis

Data were statistically analyzed using SPSS ver. 21.0. Measurement data were expressed as mean ± standard deviation (SD). Analysis of variance (ANOVA) for repeated measurement was used in the comparison within groups, and the intra-group comparison was performed using single-factor ANOVA. Enumeration data were compared using Chi-square test or Fisher extract probability method. Difference was considered statistically significant if P<0.05.

## RESULTS

### Comparison of surgical efficacy between the two groups

Children in both group successfully completed the operation. The indicators such as duration of operation, intraoperative blood loss, time to recovery of peristalsis and pain score in the observation group were superior to those of the control group, and the difference had statistical significance (P<0.05; Table-I).

**Table-I:** Comparison of surgical condition between the two groups.

*Group*	*n*	*Duration of operation (min)*	*Intraoperative blood loss (mL)*	*Time to recovery of peristalsis (h)*	*Pain score*
Observation group	108	144.9±20.2	25.8±10.1	23.0±2.6	1.1±0.3
Control group	108	168.7±23.4	75.9±19.8	35.2±4.4	3.7±0.4
t		5.913	17.491	19.387	10.324
P		<0.05	<0.05	<0.05	<0.05

### Comparison of different indicators between the two groups in perioperative period

The MAP and HR of the control group were higher than those of the observation group at different time points after operation, and the differences suggested statistical significance (P<0.05). The difference of SpO_2_ between the two groups at different time points after operation showed no statistical significance (P>0.05; Table-II).

**Table-II:** Comparison of postoperative MAP, HR and SpO_2_ between the two groups.

*Indicators*	*Group*	*T0*	*T1*	*T2*	*T3*
MAP (p/mm Hg)	Observation group	80.1±9.2	84.1±6.8*	78.0±7.0*	81.0±3.5*
	Control group	81.6±5.2	93.8±6.2^#^	91.3±4.2^#^	89.7±4.8^#^
HR (p/mm Hg)	Observation group	114.8±13.1	108.2±16.3*	101.8±15.1*	97.0±11.2*
	Control group	116.6±13.8	143.7±8.8^#^	132.0±11.2^#^	124.2±13.0^#^
SpO_2_ (p/mm Hg)	Observation group	97.7±0.9	99.2±1.1	98.6±0.8	99.0±1.0
	Control group	98.2±1.1	95.8±2.0	98.0±1.9	99.0±1.0

* ***Note:*** indicated P<0.05 compared to the control group;

# indicated P<0.05 compared to T0.

### Comparison of postoperative complications between the two groups

In the observation group, there were 2 cases of enterocolitis, two cases of intestinal perforation and three cases of intestinal obstruction after operation; the incidence of adverse reaction was 6.5%. In the control group, there were 7 cases of incision infection, three cases of disruption of wound, two cases of enterocolitis, three cases of enterobrosis and 6 cases of intestinal obstruction, the incidence of adverse reaction was 19.4%. The difference of incidence of adverse reactions between the two groups had statistical significance (X^2^=4.797; P<0.05).

### Comparison of follow up results between the two groups

**Fig.1:**
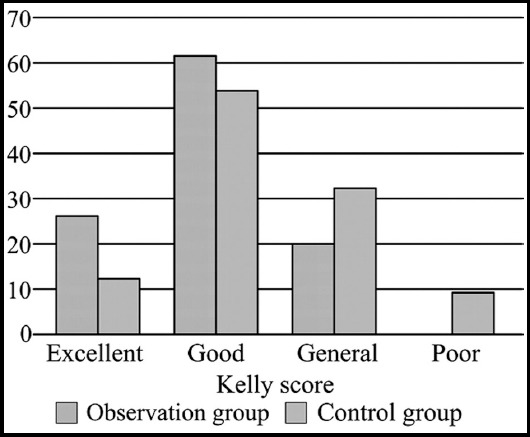
Comparison of Kelly score between the two groups

Two hundred and sixteen children were all successfully followed up (100%). The follow up lasted for six months to one year. In the period, the children had normal defecate character and reduced defecation frequency. The excellent and good rate of Kelly score of the observation group was 81.5%, much higher than 61.1% in the control group, and the difference had statistical significance (P<0.05; Fig.1).

## DISCUSSION

Hirschsprung’s disease is usually located at distant intestinal canal. Intestinal peristalsis weakens because of ganglion cell function loss in distant intestinal canal, which can induce intestinal canal spasm and limit normal physiological activity. Once developing Hirschsprung’s disease, children are unable to normally defecate by themselves, and moreover the inhibited nutritional absorption can severely affect the health growth of children. A study has suggested that, Hirschsprung’s disease children with lower age have a lower incidence of secondary intestinal tract lesion,^8^ which could improve radical effect and was beneficial to the recovery of postoperative defecation function. The authors of this study advocate early diagnosis and treatment. Besides the aforementioned reason, early treatment also has the following benefits. Firstly, time waiting for operation can be shortened, which can relieve the economic and psychological burdens. Secondly, the operative difficulty is low. Infants have thin mesocolon, no fat and clear mesenteric vessels; hence it is easy to process mesenterium and anorectum under a laparoscope. Moreover, the ectocolon of children is mild; therefore, the intestinal canal is easy to be pulled out from the muscle sheath of the rectum. Thirdly, the postoperative defecation function of children recovers rapidly, the incidence of complications such as enterocolitis is low, and younger children have stronger adaption and shaping ability compared to elderly children.

Treatment of Hirschsprung’s disease has witnessed a series of development and evolution.^9^,^10^ With the constant development of medical technology in recent years, laparoscope has been an important diagnosis and treatment method for minimally invasive surgery.^11^,^12^,^13^ Laparoscopic modified Soave operation can provide a wide field; surgical doctors can observe all corners of the abdominal cavity to confirm the position of lesions through the wide field, which can improve the accuracy of surgery and avoid unnecessary operative wound and cracking of wound.^14^ In 1999, Albanese CT et al.^15^ treated newborns with laparoscope in combination with radical operation and obtained favorable short-term effect. In 2001, Zheng S. et al.^16^ reported that treatment for Hirschsprung’s disease of children evolved from open surgery, laparoscopic surgery, transanal colon pull-through surgery to laparoscope in combination with transanal pull-through surgery. Besides short-segment Hirschsprung’s disease (diseased colon segment < 3 cm) which is treated by intersphincteric resection and total colonic Hirschsprung’s disease which is treated by laparotomy, other types of Hirschsprung’s disease are all treated by transanal colon pull-through surgery at first. If it is difficult to pull through colon or the resected colon segment is not enough, then laparoscope is used to dissociate blood vessels, mesenterium and splenic flexure of colon. Almost all lesions can be treated by surgery under the assistance of laparoscope.

Laparoscopic modified Soave operation changes traditional surgical approach, but maintains the principle of typical Hirschsprung’s disease radical surgery; it is featured by mild surgical invasion, small scar, fast recovery, mild disturbance to abdominal cavity and low incidence of adhesive intestinal obstruction. The application of ultrasound knife in dissociating mesenterium reduces amount of bleeding, relieve pain of children, reduce Hirschsprung’s disease induced complications, and improve the tolerance of newborns and infants to surgery. With the development of minimally invasive surgery, laparoscopic treatment has been generally accepted as a satisfactory surgery for Hirschsprung’s disease. In this study, the observation group in which children were treated by laparoscopic modified Soave operation had shorter duration of operation, less intraoperative blood loss, shorter time to recovery of peristalsis and lower postoperative Visual Analogue Scale (VAS) score compared to the observation group in which children were treated by the traditional open radical resection, fully suggesting the superiority of laparoscopic modified Soave operation. Moreover, the abdominal cavity was not exposed in air during operation, suggesting laparoscopic modified Soave operation could effectively reduce incidence of infection. The incidence of complications in the observation group was much lower than that in the control group, and the vital sign change of the observation group had milder fluctuation compared to that of the control group in perioperative period, indicating the high reliability and safety of the operation.

## CONCLUSION

Laparoscopic modified Soave operation resulted in mild trauma, small changes of vital signs in perioperative period, rapid recovery, little disturbance in the abdominal cavity and low incidence of adhesive intestinal obstruction in the treatment of Hirschsprung’s disease. Moreover, ultrasonic knife was used during operation, avoiding excessive separation of the pelvic cavity, which reduces blood loss, shortens operation time and reduce the pain of children. With the improvement of laparoscopic technology level, laparoscopic modified Soave operation will certainly replace the traditional open surgery and become the preferred scheme for the clinical treatment of Hirschsprung’s disease.

### Authors’ Contribution

**YLT & YRW:** Study design, data collection and analysis.

**TTS, FW & YRW:** Manuscript preparation, drafting and revising.

**YLT & YRW:** Review and final approval of manuscript.
